# Supplementary material for: Mobile application traffic reveals multifunctional use patterns in Parisian parks
Source: Sci Rep. 2026 Sep 8;16:28090. doi: 10.1038/s41598-026-61506-6 (PMC13554209; doi:10.1038/s41598-026-61506-6)
Supplement: Supplementary file 1 [file 41598_2026_61506_MOESM1_ESM.pdf]

1 Studied Parks

We present in Table 1 the complete set of parks included in our study, which we are able to confidently isolate and assign mobile traffic demands, following the methodology described in the methodology (Section 4.3 of the main text). We include as well the clusters they are assigned in our analysis (Section 2.3 of the main text), their area, the number of antennas with good illumination in each, their Quality of Coverage (Q_p) metric and a traffic score, which is normalized across all studied parks to give a notion of traffic volume across them.

2 Variation of RSCA on Park Clusters Across Workdays and Weekends

We observe an interesting pattern on the RSCA values across park clusters: both Lunchbreak and Recreational parks have their application preferences change on weekends, in relation to weekdays. More specifically, categories such as News, Productivity and Social, which on Lunchbreak and Cultural parks have opposite behaviors from Recreational parks, see these patterns reverse on weekends, with preference looking closer to a Recreational cluster park. This can indicate that these parks have distinct usages across the weeks, with parks within the urban center of Paris looking more like Recreational parks during the weekend (as their work-related users may not be present, leaving only residents of the area using those parks for more traditional recreational activities - as reflected in our measurements).

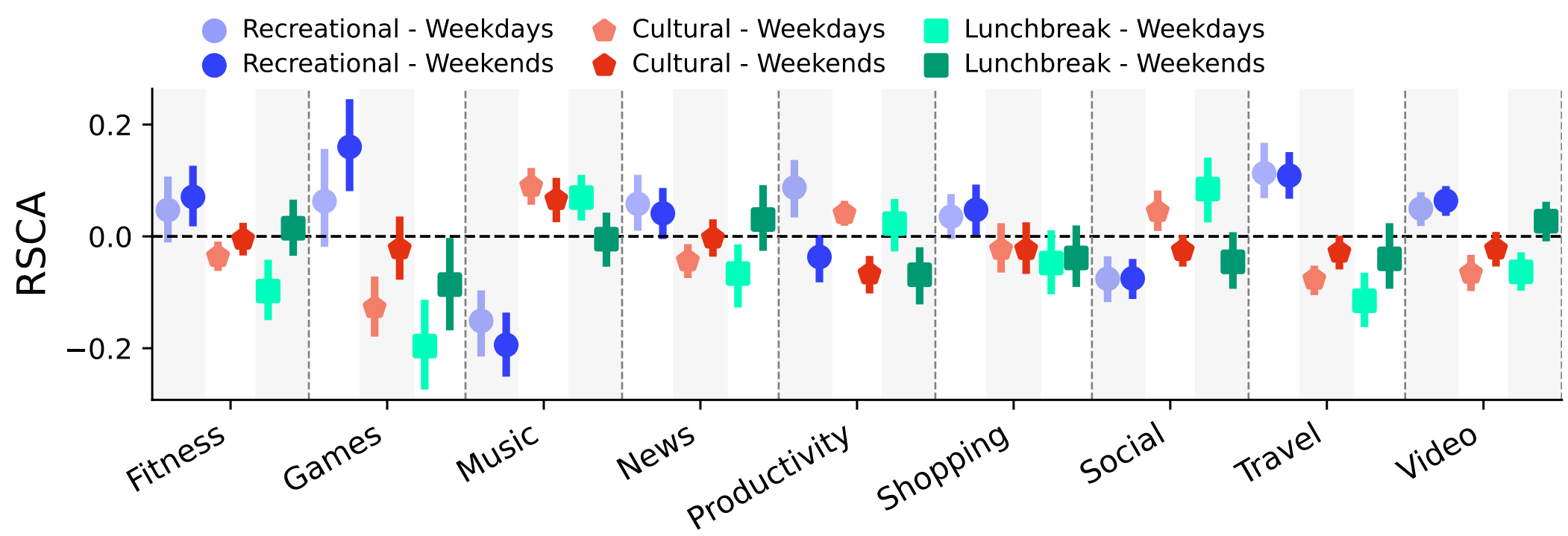

Figure 1. RSCA across apps per park category, split between weekday and weekend. Points show mean values, while lines indicate 95% confidence intervals across parks in each cluster.

3 Robustness and Sensitivity Analysis of Clustering

Stability Under Noise Perturbation. To assess the robustness of the clustering results, we conducted a noise-perturbation stability analysis. Gaussian noise was added independently to each feature, with the noise standard deviation scaled to 1%, 5%, and 10% of the empirical feature-wise standard deviation. For each noise level, spectral clustering was re-estimated with identical parameter settings. The resulting partitions were compared to the original clustering using the Adjusted Rand Index (ARI). The ARI remained equal to 1.00 under 1% noise, indicating perfect stability, and remained high (ARI ≈ 0.94) under both 5% and 10% perturbations. These results indicate that the identified park clustering is robust to moderate levels of random noise and is not driven by small fluctuations in feature values.

Sensitivity to graph density. To evaluate the sensitivity of the clustering results to the graph construction, we conducted a parameter robustness analysis by varying the number of nearest neighbors used to construct the affinity matrix. Specifically, we re-estimated the spectral clustering model with $n \in \{5, 10, 15, 20\}$ nearest neighbors, while keeping all other parameters fixed. The resulting partitions were compared to the baseline specification ($k = 10$) with ARI values equal to 0.71 for $n = 5$, 1.00 for $n = 10$, 0.81 for $n = 15$, and 0.94 for $n = 20$. These results indicate that the clustering structure is generally stable across a reasonable range of neighborhood sizes, with higher stability observed for moderate to larger values of n. While smaller neighborhood sizes ($n = 5$) introduce some variation in cluster assignments, the overall partition remains largely consistent, suggesting that the identified park typology is not highly sensitive to the specific choice of graph connectivity parameter.

Validation metrics. To further assess the appropriateness of the selected number of clusters, we compared spectral clustering across a range of cluster numbers ($k = 2$ to $k = 9$) using three internal validation metrics: Silhouette score, Calinski-Harabasz (CH) index, and Davies-Bouldin (DB) index. The Silhouette and CH indices evaluate cluster compactness and separation (higher values indicate better-defined clusters), while the DB index measures average cluster similarity (lower values indicate better separation). Results are seen on Figure 2. The Silhouette score reaches its maximum at $k = 2$ and decreases for larger

Table 1. The selected set of 45 parks and their respective clusters, with their area (in ha), the number of antennas with good coverage used for their analysis, as well as the Quality of Coverage Q_p score. We also present the normalized traffic score of each park, which adds a notion of the different volume of overall traffic at each park.

Park name	Cluster	Area (ha)	Antennas	Q_p	Traffic score
Bois de Boulogne	Cultural	836.83	39	0.85	0.3111
Bois de Vincennes	Cultural	1000.28	42	0.81	0.3679
Champ de Mars	Cultural	28.77	8	0.55	0.4804
Cité Universitaire - Parc Est	Cultural	26.74	7	0.41	0.3731
Cité Universitaire - Parc Ouest	Cultural	7.62	3	0.48	0.3562
Domaine national de Saint-Cloud	Cultural	376.95	17	0.66	0.1614
Jardin des Tuileries	Cultural	22.38	7	0.47	0.4920
Jardin du Ranelagh	Cultural	10.14	5	0.50	0.3559
Parc Georges Brassens	Cultural	7.58	2	0.55	0.5803
Parc Montsouris	Cultural	16.28	3	0.61	0.4020
Parc Pierre-Lagravère	Cultural	29.98	5	0.40	0.9471
Parc de Bercy	Cultural	14.65	4	0.53	0.5322
Parc de la Villette	Cultural	33.66	5	0.61	0.7282
Parc des Buttes-Chaumont	Cultural	25.68	8	0.54	0.3986
Parc du Lycée Michelet	Cultural	9.03	2	0.44	0.6506
Parc départemental Jean-Moulin - Les Guilands	Cultural	25.25	2	0.53	0.7790
Square des Batignolles	Cultural	1.72	2	0.47	0.5760
Jardin des Plantes	Lunchbreak	16.17	4	0.42	1.0000
Jardin du Luxembourg	Lunchbreak	22.23	7	0.60	0.3216
Jardins Abbé Pierre - Grands Moulins	Lunchbreak	0.78	1	0.59	0.3486
Jardins du Trocadéro	Lunchbreak	13.79	4	0.50	0.5438
Parc André Citroën	Lunchbreak	9.58	3	0.43	0.4966
Parc Clichy-Batignolles Martin Luther King	Lunchbreak	9.92	3	0.61	0.7398
Parc Départemental des Chanteraines	Lunchbreak	72.06	4	0.53	0.2182
Parc Floral de Paris	Lunchbreak	33.50	2	0.57	0.2543
Parc Monceau	Lunchbreak	8.43	4	0.46	0.4667
Parc Suzanne Lenglen	Lunchbreak	18.91	4	0.41	0.6661
Parc de Sceaux	Lunchbreak	96.40	6	0.69	0.5154
Arboretum de Paris	Recreational	12.98	1	0.48	0.0000
Parc Henri Sellier	Recreational	25.11	2	0.46	0.5296
Parc Interdépartemental des Sports - Plaine Sud	Recreational	107.80	5	0.59	0.0449
Parc Lefèvre	Recreational	9.03	1	0.41	0.4345
Parc Nature du Plateau d'Avron	Recreational	11.27	2	0.53	0.0299
Parc de la Butte du Chapeau Rouge	Recreational	4.74	2	0.41	0.2519
Parc de la Vallée-aux-Loups	Recreational	45.14	3	0.52	0.3782
Parc des Artistes	Recreational	9.35	2	0.52	0.1407
Parc des Saules	Recreational	6.55	2	0.42	0.0994
Parc départemental Georges-Valbon	Recreational	315.63	13	0.73	0.1187
Parc départemental de la Fosse Maussoin	Recreational	23.32	3	0.42	0.5094
Parc départemental de la Haute-Île	Recreational	74.71	5	0.42	0.1999
Parc départemental de la Plage Bleue	Recreational	40.99	2	0.42	0.4009
Parc départemental des Lilas	Recreational	83.93	8	0.55	0.1531
Parc départemental du Sausset	Recreational	205.30	9	0.75	0.1104
Parc intercommunal des Sports du Grand Godet	Recreational	21.53	3	0.53	0.1094
Île de Loisirs de Créteil	Recreational	45.86	7	0.56	0.3693

values of k, indicating that additional clusters reduce average separation in Euclidean space. A similar declining pattern is observed for the CH index. The DB index fluctuates across specifications and does not exhibit a pronounced global minimum at higher k - however, its minimum is achieved at $k = 3$. Taken together, the three metrics consistently indicate stronger

Table 2. Relative importance of Flickr tags within the three identified groups of parks. The values after the tag indicate how much more frequent these tags were compared to a uniform distribution (Section 4.8 of the main text).

	Lunchbreak Parks	Cultural Parks	Recreational Parks
1	plant, 2.73	bercy, 3.72	beach, 3.84
2	arbre, 2.19	music, 2.32	arbre, 3.66
3	architecture, 2.13	sculpture, 1.82	iledefrance, 3.17
4	text, 2.01	festival, 1.68	automne, 2.57
5	face, 2.01	live, 1.66	art, 1.83
6	tree, 2	concert, 1.61	spring, 1.73
7	water, 1.83	iphoneography, 1.51	tree, 1.69
8	film, 1.79	instagramapp, 1.51	fleurs, 1.59
9	jardin, 1.72	tree, 1.48	nature, 1.59
10	garden, 1.7	fleurs, 1.44	fleur, 1.27
	automne, 1.66; people, 1.61; iphone, 1.55; bercy, 1.48; outdoor, 1.46; blue, 1.41; fleurs, 1.39; urban, 1.39; iledefrance, 1.34; autumn, 1.32; instagramapp, 1.31; iphoneography, 1.3; beach, 1.29; landscape, 1.29; mountain, 1.25; flower, 1.24; trees, 1.22; street, 1.17; statue, 1.15; geotagged, 1.14; nature, 1.13; sculpture, 1.12; car, 1.1; museum, 1.09; flowers, 1.08; winter, 1.07; nuit, 1.07; spring, 1.03; white, 1.01	text, 1.37; automne, 1.35; seine, 1.27; face, 1.25; geotagged, 1.22; architecture, 1.21; art, 1.2; arbre, 1.2; iphone, 1.19; street, 1.18; water, 1.16; iledefrance, 1.09; plant, 1.09; outdoor, 1.08; spring, 1.06	

cluster separation for smaller values of k, with $k = 3$ representing a reasonable balance between cluster compactness and interpretability. Importantly, while internal validation metrics naturally decline as cluster granularity increases, the overall patterns are consistent, supporting the stability and structural coherence of the identified park classes.

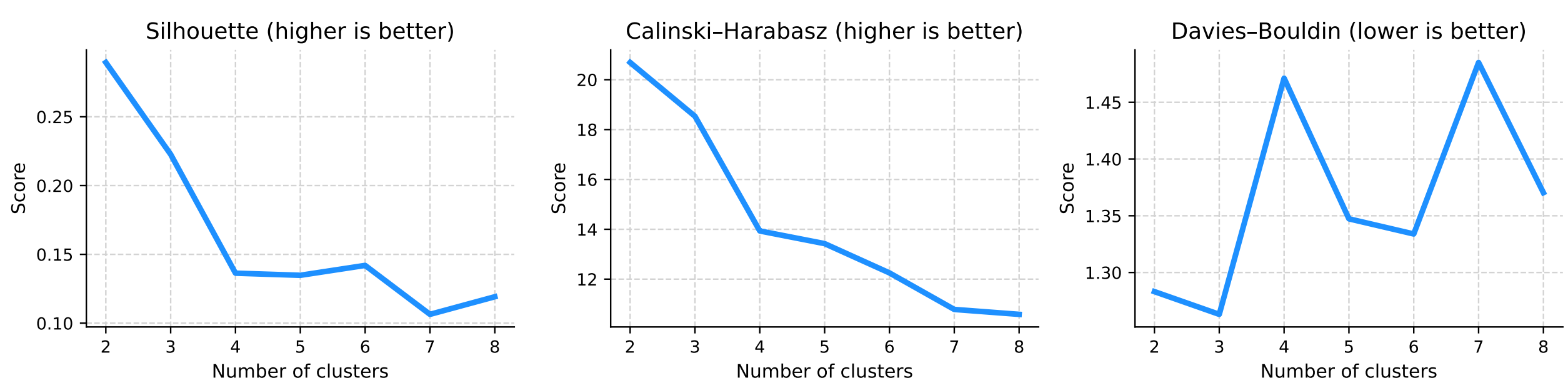

Figure 2. Comparison of internal clustering validation metrics for spectral clustering and k-means across $k = 2$–9. Silhouette and Calinski–Harabasz scores (higher values indicate better separation) and Davies–Bouldin index (lower values indicate better separation) are reported to assess cluster compactness and distinctiveness.

4 Image Sharing Varies Among Park Types

To provide an alternative perspective and assess whether shared images support the functional roles of parks identified through traffic data, we analyzed geo-located images from Flickr. These images were collected via the Flickr API in 2015 and include 539,763 photos across the parks in our study, an average of 12,851 images per park. Each image could be tagged with an arbitrary number of labels, both user-generated and algorithmically assigned[1,2]. On average, each image has 7.45 tags, resulting in a total of 4,022,720 tags, approximately 95,779 per park. To measure the relative importance of each tag, we calculated the ratio between its actual and expected frequency. Expected frequencies were estimated using a uniform distribution of tag occurrences across parks (see Section 4.8 of the main text for details). A relative importance value of 1 indicates an average

occurrence, while values above 1 suggest the tag appears more often than expected. The relative importance of tags for all parks in each cluster is shown in Table 2. Tags are listed in descending order of importance within each cluster, with a cutoff value of 1. The values following each tag represent the mean relative importance in that cluster.

Lunchbreak parks show the highest number of tags occurring above expectation (39), highlighting a diverse image set. Tags relate both to natural elements (e.g., 'plant', 'arbre'—French for tree, 'garden') and to urban features, such as 'architecture', 'text' (indicating signage), and 'face', which signals the presence of people in photos.

Cultural parks are characterized by tags associated with arts, concerts, and festivals. The presence of 'instagramapp' suggests that many images were also shared on Instagram. Overall, the tag patterns reinforce the cultural and touristic appeal of these parks.

Recreational parks have the fewest tags above expectation ($n = 10$), reflecting lower tag diversity. Many of these tags are in French, suggesting that the user-contributed tags come from a predominantly local user base.

Predictive Power of Flickr Tags. To quantitatively assess the relevance of tag frequency, we conducted a prediction experiment to determine whether Flickr tags can be used to classify the functional uses of parks. We implemented a 3-fold cross-validated Random Forest classifier with 100 estimators, using a feature vector representing the relative importance of the 25 most frequent tags per park. The prediction target was the park's assigned cluster. Our model achieved accuracies ranging between $[0.571; 0.715]$, with a mean accuracy of 0.643 and an F_1 score of 0.619. Our model performs significantly better than chance (0.333), confirming that images shared in parks with different functional uses exhibit distinct patterns. However, its predictive power is only moderate. This is likely because Flickr tags alone are insufficient for reliably classifying park functions, due to the noisy nature of tagging data and imbalances in the number of photos per park. Additionally, there is a temporal gap of approximately 10 years between the two datasets.

5 Supplementary Tables

We next describe additional statistical tests performed for the insights gained throughout Section 2 of the main text. The first test seeks to understand if the distribution of RSCA values across application categories differs within all parks in Paris, versus the remaining areas of the city. We first use a Levene's test to assess if the distributions' variances are equal. With this result, we select a t-test to validate if the distributions are statistically different. A Levene p-value below 0.05 leads to a Welch's t-test as the distributions have unequal variances, while a p-value above 0.05 leads to a Student's t-test (as we can assume the distributions have equal variances). Through the t-tests, we find that 4 application categories have a significant statistical difference in their preferences in parks versus the rest of the city: Games and News have $p < 0.05$ and Music and Social have $p < 0.01$. All results are detailed in Table 3.

Table 3. T-test results assessing significant differences in per-category RSCA between parks and the city. Bold values represent that there is a significant difference between park and city, while asterisks represent the intensity (* for $p < 0.05$, ** for $p < 0.01$). A Levene's test was first applied to check variance similarity, in order to select the correct t-test.

Application Category	Levene p-value	Selected t-test	p-value
Fitness	0.104	Student's	0.214
Games	0.025	Welch's	**0.027***
Music	0.907	Student's	**0.005****
News and Info	0.344	Student's	**0.049***
Productivity	0.549	Student's	0.112
Shopping	0.004	Welch's	0.086
Social	0.491	Student's	**0.005****
Travel	0.194	Student's	0.164
Video	0.840	Student's	0.077

Next, we replicate the previous experiment but with park clusters, to understand if each cluster has statistically significant application preference versus the rest of the city. The full results are presented in Table 4.

Finally, we explore for each park cluster if there is a significant correlation between application preference and socioeconomic indicators. We calculate the Pearson correlation between values, and consider a correlation statistically relevant if p<0.05. Results are fully detailed in Table 5, with results discussed in Section 2.4 of the main text.

Table 4. T-test results assessing significant differences in per-category RSCA between parks and the city. Bold values represent that there is a significant difference between park and city, while asterisks represent the intensity (* for $p < 0.05$, ** for $p < 0.01$). A Levene's test was first applied to check variance similarity, in order to choose the correct t-test.

Application Category	Cluster	Levene p-value	Selected t-test	t-test p-value
Fitness	Recreational	0.820	Student	**0.029***
	Cultural	0.010	Welch	0.711
	Lunchbreak	0.347	Student	0.351
Games	Recreational	0.602	Student	0.052
	Cultural	0.016	Welch	**0.048***
	Lunchbreak	0.256	Student	**0.040***
Music	Recreational	0.314	Student	**0.004****
	Cultural	0.005	Welch	**<0.001****
	Lunchbreak	0.044	Welch	**0.007****
News	Recreational	0.284	Student	**0.017***
	Cultural	0.011	Welch	0.956
	Lunchbreak	0.496	Student	0.929
Productivity	Recreational	0.003	Welch	**0.013***
	Cultural	<0.001	Welch	0.063
	Lunchbreak	0.009	Welch	0.149
Shopping	Recreational	0.537	Student	**0.020***
	Cultural	0.481	Student	0.656
	Lunchbreak	0.053	Student	0.196
Social	Recreational	0.678	Student	**<0.001****
	Cultural	0.683	Student	0.856
	Lunchbreak	0.145	Student	0.342
Travel	Recreational	0.440	Student	**<0.001****
	Cultural	0.002	Welch	0.054
	Lunchbreak	0.285	Student	0.099
Video	Recreational	0.769	Student	**<0.001****
	Cultural	0.067	Student	**0.009****
	Lunchbreak	0.309	Student	0.368

References

1. Li, L.-J., Socher, R. & Fei-Fei, L. Towards total scene understanding: Classification, annotation and segmentation in an automatic framework. In *Conference on Computer Vision and Pattern Recognition*, 2036–2043, DOI: 10.1109/cvpr.2009.5206718. IEEE (IEEE, 2009).

2. Thomee, B. *et al.* Yfcc100m: The new data in multimedia research. *Commun. ACM* **59**, 64–73, DOI: 10.1145/2812802 (2016).

Table 5. Correlation statistics and p-values for socioeconomic indicators across park categories and selected application categories. Bold values indicate statistically significant correlations ($p \leq 0.05$).

	Median Income		
**Indicator**	**Cultural parks**	**Lunchbreak parks**	**Recreational parks**
Games	0.093 (0.723)	-0.273 (0.416)	**-0.672 (0.003)**
Music	0.322 (0.208)	**0.728 (0.011)**	**0.608 (0.010)**
News and Information	**0.606 (0.010)**	-0.092 (0.788)	0.317 (0.215)
Social	-0.188 (0.471)	0.254 (0.450)	-0.067 (0.798)

	Gini Index		
**Indicator**	**Cultural parks**	**Lunchbreak parks**	**Recreational parks**
Games	0.153 (0.559)	-0.259 (0.442)	0.264 (0.306)
Music	0.295 (0.250)	**0.691 (0.018)**	-0.035 (0.893)
News and Information	**0.697 (0.002)**	0.060 (0.861)	0.213 (0.412)
Social	-0.007 (0.979)	0.293 (0.383)	-0.212 (0.414)

	Distance		
**Indicator**	**Cultural parks**	**Lunchbreak parks**	**Recreational parks**
Games	-0.063 (0.810)	0.392 (0.233)	0.379 (0.134)
Music	-0.449 (0.070)	-0.318 (0.340)	-0.394 (0.118)
News and Information	-0.212 (0.415)	0.086 (0.801)	-0.234 (0.365)
Social	-0.121 (0.645)	-0.560 (0.073)	-0.038 (0.884)

	Park size		
**Indicator**	**Cultural parks**	**Lunchbreak parks**	**Recreational parks**
Games	**0.589 (0.013)**	0.487 (0.129)	**0.608 (0.010)**
Music	0.466 (0.059)	0.091 (0.790)	-0.095 (0.716)
News & Information	0.147 (0.574)	-0.070 (0.839)	0.326 (0.202)
Social	-0.317 (0.216)	-0.555 (0.076)	-0.063 (0.811)
